# Cost-Effectiveness Analysis of Universal Vaccination of Adults Aged 60 Years with 23-Valent Pneumococcal Polysaccharide Vaccine versus Current Practice in Brazil

**DOI:** 10.1371/journal.pone.0130217

**Published:** 2015-06-26

**Authors:** Patrícia Coelho de Soárez, Ana Marli Christovam Sartori, Angela Carvalho Freitas, Álvaro Mitsunori Nishikawa, Hillegonda Maria Dutilh Novaes

**Affiliations:** 1 Departamento de Medicina Preventiva, Faculdade de Medicina da Universidade de São Paulo, São Paulo, SP, Brazil; 2 Clínica de Moléstias Infecciosas e Parasitárias, Hospital das Clínicas da Faculdade de Medicina da Universidade de São Paulo, São Paulo, SP, Brazil; Instituto Butantan, BRAZIL

## Abstract

**Objective:**

To evaluate the cost-effectiveness of introducing universal vaccination of adults aged 60 years with the 23-valent pneumococcal polysaccharide vaccine (PPV23) into the National Immunization Program (NIP) in Brazil.

**Methods:**

Economic evaluation using a Markov model to compare two strategies: (1) universal vaccination of adults aged 60 years with one dose of PPV23 and 2) current practice (vaccination of institutionalized elderly and elderly with underlying diseases). The perspective was from the health system and society. Temporal horizon was 10 years. Discount rate of 5% was applied to costs and benefits. Clinical syndromes of interest were invasive pneumococcal disease (IPD) including meningitis, sepsis and others and pneumonia. Vaccine efficacy against IPD was obtained from a meta-analysis of randomized control trials and randomized studies, whereas vaccine effectiveness against pneumonia was obtained from cohort studies. Resource utilization and costs were obtained from the Brazilian Health Information Systems. The primary outcome was cost per life year saved (LYS). Univariate and multivariate sensitivity analysis were performed.

**Results:**

The universal vaccination strategy avoided 7,810 hospitalizations and 514 deaths, saving 3,787 years of life and costing a total of USD$31,507,012 and USD$44,548,180, respectively, from the health system and societal perspective. The universal immunization would result in ICERs of USD$1,297 per LYS, from the perspective of the health system, and USD$904 per LYS, from the societal perspective.

**Conclusion:**

The results suggest that universal vaccination of adults aged 60 years with the 23-valent pneumococcal polysaccharide vaccine (PPV23) is a very cost-effective intervention for preventing hospitalization and deaths for IPD and pneumonia is this age group in Brazil.

## Introduction

*Streptococcus pneumoniae*-associated infections are a leading cause of morbidity and mortality worldwide, particularly in low- and middle-income countries. Under-two children, adults aged >60 years and persons with chronic conditions are the most affected. [1]

The 23-valent pneumococcal polysaccharide vaccine (PPV23) has shown efficacy against invasive pneumococcal disease (IPD) in adults and has been widely used in developed countries. [2, 3] In developing countries, PPV23 use has not been considered a priority. Doubts regarding the benefits of PPV23 have limited its use among the elderly. In the Brazilian National Immunization Program (NIP), PPV23 has been incorporated into the immunization schedule of institutionalized elderly and persons aged >2 years with chronic conditions since 1999 [4], however data on vaccine coverage in this population is scarce. [5] In 2010, the 10-valent pneumococcal conjugate vaccine (PCV10) has been introduced into childhood immunization schedule (4 doses, at 2, 4, 6 and 15 months). Catch-up for children in the second year of life with single dose was conducted in the first year of the program. The nationwide overall coverage of PCV10 increased from 81.7%, in 2011, to 92.7, in 2013, with regional variations.

In Brazil, since 2005, health technology assessments and economic evaluation studies have been requested for evaluating the introduction of new vaccines into the NIP. These studies evaluated seven new vaccines (rotavirus, PCV10, meningococcal C conjugate, varicella, hepatitis A, inactivated polio and HPV), all them recently incorporated in the Public Health System (*Sistema Único de Saúde*, *SUS*).

The objective of this study is to evaluate the cost-effectiveness of introducing universal vaccination of adults aged 60 years with the PPV23 into the NIP in comparison to the current practice (target vaccination of high-risk persons aged >2 years with chronic conditions and institutionalized elderly), within the context of the SUS in Brazil, as requested by the NIP General Coordination / Ministry of Health.

## Materials and Methods

### Decision analysis model

**Fig 1:**
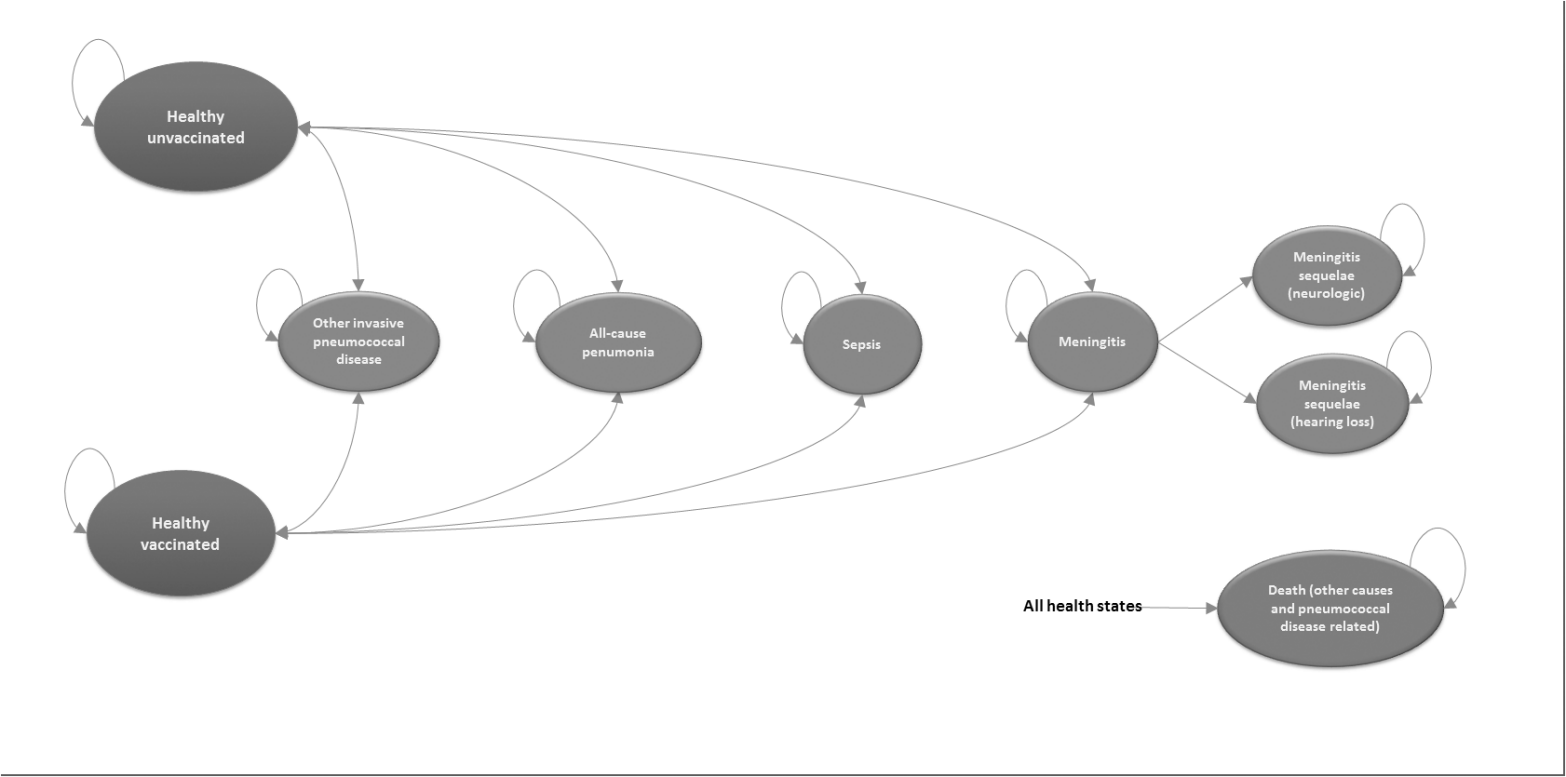
Influence diagram showing the structure of the Markov model of cost-effectiveness analyses of implementing universal vaccination program with 23-valent polysaccharide pneumococcal vaccine (PPV23) for persons aged 60 years in Brazil. Base case scenario included IPD (G.001, A40.3, and B95.3) and all-cause pneumonia (J12.0 to J18.9). The first structural sensitivity analysis included IPD (G.001, A40.3, and B95.3), and no vaccine effect against pneumonia; the second structural sensitivity analysis included IPD (G.001, A40.3, and B95.3), and pneumococcal pneumonia (J13) in place of all-cause pneumonia (J12.0 to J18.9).

A Markov model was programmed using the software Microsoft Office Excel 2014. The Markov cycle length was 1 year, and was evaluated over 10 years. The model compares two strategies: (1) universal vaccination of adults aged 60 years with one dose of PPV23 and 2) current vaccination strategy (vaccination of institutionalized elderly or elderly with underlying diseases). The structure of the model considered eight health states: healthy unvaccinated, healthy vaccinated, all-cause pneumonia, pneumococcal sepsis, pneumococcal meningitis, other IPD, sequelae of meningitis and death (Fig 1).

A cohort of all 60 years old persons were included in the model. Risk-stratifying was not done. Individuals could enter the model in either a "healthy vaccinated" or "healthy unvaccinated" state. From these, they can move through the other six health states. Only patients with meningitis may move into a "sequelae of meningitis" state.

The model assumed that the individuals can progress to "death from other causes" state, in addition to pneumococcal disease or all-cause pneumonia related deaths. "Death from other causes" estimates were taken from *Instituto Brasileiro de Geografia e Estatística*, IBGE [6]. Other health states evolve to the state of death according to specific probabilities of mortality for each health state.

### Model assumptions

- In each cycle, modeled individuals were in one of the eight health states, and could not develop two diseases (e.g., pneumonia and meningitis) in the same cycle.
- In the "healthy vaccinated" state, individuals receive one dose of vaccine at time zero. VE decreases according to the estimates of loss of immunity after vaccination ("waning") (Table 1).
- All individuals who become ill return to their initial states ("healthy vaccinated" or "healthy unvaccinated") in the next cycle and have the same risk of illness of those individuals who did not have the disease in the previous cycles.
- Individuals who transitioned to the sequelae meningitis state remained there for the rest of the model period or left by death.
- Indirect protection of adults due to PCV10 children vaccination program was not considered in the model.

**Table 1:** Base case model effectiveness and epidemiological data with sensitivity analysis ranges used in the cost-effectiveness analyses of 23-valent polysaccharide pneumococcal vaccine (PPV23) for persons aged 60 years in Brazil.

	Base case	Sensitivity analysis	
Parameter	Value	Range (min—max)	Source
**Vaccination program**			
Vaccine efficacy/effectiveness			
Against IPD			[3, 7]
First 2 years	68%	53–78%	
3rd- 5th year	61.20%	46.2–71.2%	
6th- 10th year	52.70%	37.7–62.7%	
Waning protection			
3rd year	6.80%		[7]
6 th year	8.50%		[7]
Against all-cause pneumonias			
First 5 years	25%	2–42%	[11]
6th- 10th year	0%		
Against pneumococcal pneumonias			
First 5 years	45%		[12]
6th- 10th year	0%		
Vaccine coverage	80%	70–90%	NIP
Dose price	USD$6.25		NIP
5% wastage rate	USD$0.31		WHO
Administration cost	USD$1.90		[36, 37]
Total vaccine cost	USD$8.46		
**Disease burden**			
Pneumococcal meningitis incidence*			
60–64 years	0.000014	-50%- 25%—+25% +50%	[13, 15]
65–69 years	0.000012	-50%- 25%—+25% +50%	[13, 15]
Pneumococcal sepsis hospitalization *			
60–64 years	0.000083	-50%- 25%—+25% +50%	[13]
65–69 years	0.000131	-50%- 25%—+25% +50%	[13]
Other IPD (ICD-10 code B95.3) hospitalization *			
60–64 years	0.000004	-50%- 25%—+25% +50%	[13]
65–69 years	0.000005	-50%- 25%—+25% +50%	[13]
All-cause pneumonia hospitalization*			
60–64 years	0.006061	-50%- 25%—+25% +50%	[13, 15]
65–69 years	0.009068	-50%- 25%—+25% +50%	[13, 15]
Pneumococcal pneumonia hospitalization*			
60–64 years	-	0.0000261437	[11]
65–69 years	-	0.0000431651	[11]
Pneumococcal meningitis case-fatality			
60–64 years	0.41	-50%- 25%—+25% +50%	[13]
65–69 years	0.58	-50%- 25%—+25% +50%	[13]
Pneumococcal sepsis hospital case-fatality			
60–64 years	0.27	-50%- 25%—+25% +50%	[13]
65–69 years	0.39	-50%- 25%—+25% +50%	[13]
Other IPD (B95.3) hospital case-fatality			
60–64 years	0.043	-50%- 25%—+25% +50%	[13]
65–69 years	0.16	-50%- 25%—+25% +50%	[13]
All-cause pneumonia hospital case-fatality			
60–64 years	0.098	-50%- 25%—+25% +50%	[13]
65–69 years	0.108	-50%- 25%—+25% +50%	[13]
Pneumococcal pneumonia hospital case-fatality			
60–64 years	-	0.07	[11]
65–69 years	-	0.07	[11]
Sequelae after pneumococcal meningitis			
60–69 years	25.7%		[17]

IPD—Invasive pneumococcal disease

B95.3—*Streptococcus pneumoniae* as the cause of disease classified elsewhere

*These rates were converted to transition probabilities with the formula: *p* = 1 –*exp*(-*rt*). Where *p* is the transition probability, *r* is the rate, and *t* is time.

This cost-effectiveness analyses (CEA) adopted the perspective of the health system and society. The health system perspective included direct medical costs of the public health system (SUS) and private health system. The societal perspective included the direct medical costs, direct non-medical costs (transportation costs) and indirect costs of productivity losses.

The time horizon used in the analysis was 10 years, as this is the maximum accepted duration of protection afforded by PPV23. [7]

The costs were estimated in 2011 Brazilian Reais (R$) and are presented in United States Dollars (USD$) as of December 2011, at the exchange rate of USD$1 = R$1.88 [8]. All costs and effects are presented in disaggregated and aggregated form, and incremental cost-effectiveness ratios (ICERs) are presented for the primary outcome, cost per life year saved (LYS). So, only averted mortality was considered. A 5% discount rate was applied to both future costs and future health benefits, as suggested by the Brazilian guidelines [9]. The reporting of this economic evaluation followed the 2013 Consolidated Health Economic Evaluation Reporting Standards guidelines [10]. This study was approved by the Research Ethics Committee of Faculdade de Medicina da Universidade de São Paulo.

We conducted literature review and used National Health Information System databases to inform model parameters. The model parameters for vaccine effectiveness, epidemiological and cost are summarized in Tables 1 and 2.

**Table 2:** Costs data for base case model and sensitivity analysis ranges used in cost-effectiveness analyses of 23-valent polysaccharide pneumococcal vaccine (PPV23) for persons aged 60 years in Brazil (in 2011 USD$).

	Base case	Sensitivity analysis	
Parameter	Value	Range (min-max)	Source
**Hospitalization costs**			
Pneumococcal meningitis			
60–64 years	693.63	411.91–1,598.21	[13]
65–69 years	579.19	411.91–2,219.05	[13]
Pneumococcal sepsis			
60–64 years	692.55	460.59–4,206.76	[13]
65–69 years	706.29	460.59–4,381.35	[13]
Other IPD (ICD-10 code B95.3)			
60–64 years	172.67	155.65–241.01	[13]
65–69 years	215.29	172.67–1,156.27	[13]
All-cause pneumonia			
60–64 years	322.56	309.80–603.80	[13]
65–69 years	326.82	309.80–593.78	[13]
Pneumococcal pneumonia			
60–64 years	326.82	309.80–767.94	[13]
65–69 years	326.82	309.80–634.90	[13]
**Transport (sick individual and caregiver)**			
Pneumococcal meningitis	22.02		[20], Assumption
Pneumococcal sepsis	26.91		[20], Assumption
Other IPD (B95.3)	26.91		[20], Assumption
Pneumonia	22.02		[20], Assumption
**Productivity losses**			
**Sick individual**			
Pneumococcal meningitis	96.57	-50%- 25%- 25% +50%	[14], Assumption
Pneumococcal sepsis	114.13	-50%- 25%- 25% +50%	[14], Assumption
Other IPD (B95.3)	114.13	-50%- 25%- 25% +50%	[14], Assumption
Pneumonia	96.57	-50%- 25%- 25% +50%	[14], Assumption
**Caregiver**			
Pneumococcal meningitis	67.23	-50%- 25%- 25% +50%	[14], Assumption
Pneumococcal sepsis	79.45	-50%- 25%- 25% +50%	[14], Assumption
Other IPD (B95.3)	79.45	-50%- 25%- 25% +50%	[14], Assumption
Pneumonia	67.23	-50%- 25%- 25% +50%	[14], Assumption

IPD—Invasive pneumococcal disease

B95.3: *Streptococcus pneumoniae* as the cause of disease classified elsewhere

USD$1 = R$1.88 [8]

### Vaccine efficacy/effectiveness

The vaccine efficacy against IPD was obtained from a meta-analysis of 18 randomized control trials and 7 non randomized studies evaluating the PPV23 in adults [3]. Vaccine effectiveness against all-cause and pneumococcal pneumonia were obtained from two cohort population based studies [11, 12]. The duration of protection and waning immunity were obtained from a case-control study [7]. Vaccine coverage was estimated based on influenza vaccine coverage among persons aged >60 years in Brazil in 2011.

### Epidemiological and disease burden estimates

Clinical outcomes of interest were IPD, including pneumococcal meningitis, pneumococcal sepsis and other IPD, and all-cause pneumonia and pneumococcal pneumonia. The search in the 2011 health information systems databases used the International Classification of Diseases (ICD-10) codes: pneumococcal meningitis (G.001); pneumococcal sepsis (A40.3); *Streptococcus pneumoniae* as the cause of disease classified elsewhere (B95.3); pneumococcal pneumonia (J13) and all-cause pneumonia (J12.0 to J18.9), as primary diagnosis. Our base case scenario included IPD (G.001, A40.3, and B95.3) and all-cause pneumonia (J12.0 to J18.9). The first structural sensitivity analysis included IPD (G.001, A40.3, and B95.3), and no vaccine effect against pneumonia; the second structural sensitivity analysis included IPD (G.001, A40.3, and B95.3), and pneumococcal pneumonia (J13) in place of all-cause pneumonia (J12.0 to J18.9). When we considered pneumococcal pneumonia (J13), vaccine effectiveness did not consider serotype distribution, because we used systematic review for vaccine effectiveness. To consider serotype coverage in our model, we would have to consider also the serotypes coverage in the place where the vaccine effectiveness data were collected.

Age and syndrome-specific hospitalizations rates for IPD and pneumonia were retrieved from the Hospitalization Information System (*Sistema de Informações Hospitalares do SUS*, *SIH-SUS*) [13], a reliable national public administrative database. SIH-SUS registers only hospital admissions at the SUS. The number of hospital admissions in the private health system was based on the 2008 Health Supplement of the National Survey of Household Samples (*Pesquisa Nacional por Amostra de Domicílios*, *PNAD*) [14], which estimated the SUS and the private health system as responsible for, respectively, 71.7% and 28.3% of hospitalizations of persons aged 60 to 69 years for clinical causes.

Pneumococcal meningitis incidence rate was calculated based on the number of cases reported to the Notifiable Diseases Information System (*Sistema de Informação de Agravos de Notificação*, *SINAN*) [15] and population of 60-year-old Brazilian adults provided by the Brazilian Institute of Geography and Statistics (*Instituto Brasileiro de Geografia e Estatística*, *IBGE*) [16]. Unspecified bacterial meningitis (UBM) accounted for 42% of all bacterial meningitis among persons aged >60 years reported to SINAN, and pneumococcus was responsible for 40% of all bacterial meningitis with known etiology in this age group. Assuming the same proportion of pneumococcus among cases of UBM, we included in our estimates presumable cases of pneumococcal meningitis reported as UBM. This approach was also used for sepsis, since unspecified sepsis accounted for 64% of all sepsis cases among persons aged >60 years registered in SIH-SUS, and pneumococcus was responsible for 10% of all sepsis with known pathogen in this age group.

We employed a mathematical formula to convert the rate of events into a transition probability: *p* = 1 –*exp* (-*rt*) (*p* = transition probability, *r* = rate over time *t*).

Age and syndrome-specific case-fatality rates were estimated based on confirmed deaths and cases registered in SIH-SUS (sepsis, pneumonia and other) and SINAN (meningitis). Frequency of neurological sequelae (hearing loss and cognitive impairment) after pneumococcal meningitis were obtained from a systematic review and meta-analysis [17].

### Health services utilization and costs for the treatment of pneumococcal disease

In line with the perspectives adopted, the model included the following cost components: hospital care in public and private health systems, transport and productivity losses. These components capture the main costs associated with inpatient treatment of IPD and pneumonia. Cost data are shown in Table 2 and are described in more detail below.

### Universal vaccination program

The vaccine dose price was informed by the Brazilian NIP (USD$6.26). A 5% wastage rate (USD$0.31) and administration cost (USD$1.90) was added to this figure, totalizing USD$8.47 per vaccinee. In Brazil, the great majority of immunizations are delivered via the public sector.

The base case considered a single dose of vaccine given at age 60 years. The vaccine would be administered simultaneously to influenza vaccine, already included in the elderly immunization schedule, with no need for an additional visit.

The costs of the current vaccination program (institutionalized elderly and those with underlying conditions) was not considered in the analysis, because the number of PPV23 doses administered are recorded aggregated for age (all people aged >60 years) and data regarding the number of doses administered to persons aged 60 to 69 years were not available.

Costs of adverse events (AE) following VPP-23 vaccine have not been considered, since AE are rare and lightweight and the associated costs may be considered insignificant, as assumed by other authors in CEA of elderly immunization with PPV23 [18, 19].

### Hospital care

We assumed that all episodes of IPD including pneumococcal meningitis, pneumococcal septicemia and *S*. *pneumoniae* as the cause of disease classified elsewhere were hospitalized. Only hospitalized pneumonias were included in the model.

Hospitalizations costs in the SUS were obtained from *SIH-SUS* databases [13]. To account for the skewed distribution of hospitalization costs we used the median annual cost. In the sensitivity analysis, the costs were varied from its 10^th^ percentile to its 90^th^ percentiles values. There is no available data on hospitalization costs in the Brazilian private health system and we applied the same values of hospitalization as in the public system.

When the analysis was conducted from the health system perspective, the value of two outpatient medical visits was added to the hospitalization costs. When the analysis was performed from the societal perspective, two outpatient medical visits, transportation cost and the indirect cost related to productivity losses of economically active elderly and their caregivers were included.

Transportation costs were estimated based on the average fare of public transportation in the Brazilian state capitals (USD$1.23) obtained from the National Association of Urban Transport (*Associação Nacional de Transportes Públicos–ANTP*) [20]. A round trip (USD$2.46) was multiplied by the number of times that individuals and their caregivers came to receive care in the health system.

Productivity losses represent the working days lost by patient and caregiver for treating pneumococcal disease. The average length of the disease (the average length of stay in hospital according to clinical syndrome plus seven days) was multiplied by the daily income of the patient and caregiver and weighted by the proportion of the economically active population [21].

### Sensitivity analysis

We performed multiple sensitivity analyses to test the influence of parameter uncertainty and structural uncertainty on the economic outcomes of this model. First, we performed univariate sensitivity analyses evaluating changes in VE, disease incidence, treatment costs, vaccine costs, and discounting rates. Secondly, we tested the structure of the model, assuming that PPV23 does not protect against pneumonia, followed by considering pneumococcal pneumonia instead of all-cause pneumonia. Lastly, multivariate sensitivity analyses (best and worst case scenarios) were performed.

## Results

### Base case

The base case followed a cohort of individuals aged 60 years, for 10 years, and estimated that the universal immunization strategy avoided; 7,810 hospitalizations, 514 deaths, saving 3,787 years of life and costing a total of USD$31,507,012 and USD$44,548,180, respectively, from the health system and societal perspective. The discounted ICER was USD$629 per hospitalization avoided, USD$9,556 per death avoided, and USD$1,297 per life year saved (LYS) the health system perspective. From the societal perspective, universal immunization would result in a discounted ICERs of USD$438 per hospitalization avoided, USD$6,659 per death avoided, and USD$904 per LYS. (Table 3) Considering WHO criteria [22] that states that a technology should be considered highly cost-effective if the ICER is less than gross domestic product (GDP) per capita of that country (2011 Brazilian GDP per capita of USD$11,305); cost-effective (if the ICER is between one and three times GDP per capita); and not cost-effective (if the ICER is more than three times GDP per capita), universal immunization of persons aged 60 years with PPV23 is a very cost-effective strategy.

**Table 3:** Expected cumulative effects and costs of universal vaccination of persons aged 60 years with 23-valent pneumococcal polysaccharide vaccine (PPV23), ten years after program implementation in Brazil.

	Perspective			
	Health System		Societal	
Parameter	Current practice^1^	Universal immunization	Current practice^1^	Universal immunization
**Disease impact**				
N° of hospitalizations for IPD and pneumonia	72,950	65,140	72,950	65,140
N° of hospitalizations avoided		7,810		7,810
Reduction in N° of hospitalizations, %		10.7		10.7
N° of deaths^2^	139,362	138,848	139,362	138,848
N° of deaths avoided^2^		514		514
Reduction in N° of deaths, %		0.4		0.4
N° of life years	10,643,650	10,647,437	10,643,650	10,647,437
N° of life years saved		3,787		3,787
Increase in the N° of life years saved, %		0.04		0.04
**Costs** ^**3**^				
Disease treatment cost	26,593,649	23,750,772	41,124,469	36,791,940
Disease treatment cost avoided		2,842,877		4,332,528
Reduction in disease costs, %		10.7		10.5
Intervention cost^4^		7,756,240	0	7,756,240
**Total Cost**	26,593,649	31,507,012	41,124,469	44,548,180
**Incremental Total Cost**		4,913,363		3,423,712
Increase in total cost %		18.5		8.3
**Incremental Cost-effectiveness ratio—ICER**				
Cost per hospitalization avoided		629		438
Cost per death avoided		9,556		6,659
Cost per life year saved		1,297		904

^1^ Current practice: vaccination of institutionalized elderly and elderly with underlying diseases

^2^ The number of deaths included deaths from invasive pneumococcal disease (IPD) and from all-cause pneumonia. The number of deaths prevented refers to the impact of the vaccine on IPD and all-cause pneumonia.

^3^ In United States Dollars (USD$) as of December 2011.

^4^ Intervention cost includes the cost of one vaccine dose (USD$6.25), administration costs (USD$1.90 per dose) and 5% wastage of vaccine (USD$0.31)

### Sensitivity analysis

Sensitivity analyses are presented in Table 4. The results demonstrated that among the seven different varied parameters, the ICER was most sensitive to variation in vaccine effectiveness. When the model considered the lower vaccine effectiveness, the ICER had an increase of 717% (USD$1,297 to USD$10,597), but the strategy is still very cost-effective in both analytical perspectives.

**Table 4:** Sensitivity analysis of universal vaccination of persons aged 60 years with 23-valent pneumococcal polysaccharide vaccine (PPV23) in Brazil: Incremental cost-effectiveness ratio (ICERs) according to perspective.

Sensitivity analysis	Cost per life year saved^1^	
	Health System	Societal
**Univariates**		
**Base case**	**1,297**	**904**
Vaccine efficacy/effectiveness (low)	10,597	10,310
Vaccine efficacy/effecttiveness (high)	522	119
Hospitalization rate (-50%)	3,323	2,929
Hospitalization rate (+50%)	623	230
Hospitalization rate (-25%)	1,972	1,578
Hospitalization rate (+25%)	892	499
Case-fatality (-50%)	3,066	2,135
Case-fatality (+50%)	821	572
Case-fatality (-25%)	1,823	1,270
Case-fatality (+25%)	1,006	701
Hospitalization cost (percentil 90)	279	Cost saving^2^
Hospitalization cost (percentil 10)	1,379	985
Indirect cost (-50%)	NA	1,100
Indirect cost (+50%)	NA	707
Indirect cost (-25%)	NA	1,002
Indirect cost (+25%)	NA	805
Vaccine cost (-50%)	274	Cost saving^2^
Vaccine cost (-25%)	784	392
Discount rate (0%)	831	505
Discount rate (10%)	1,913	1,444
**Structural**		
Vaccine effectiveness 0% against pneumonia	12,625	12,418
Only pneumococcal pneumonia^3^	12,137	11,915
**Multivariates**		
Best scenario^4^	65	Cost saving^2^
Worst scenario^5^	48,995	48,351

NA: not applicable

^1^ In United States Dollars (USD$) as of December 2011.

^2^ More effective and less expensive strategy

^3^ Considering pneumonias that were defined as pneumococcal pneumonia in the SIH: probability of hospitalization at 60–64 years 0.00261437%, and at 65–69 years 0.00431651%; lethality 7%; hospitalization cost USD$326.82; and vaccine efficacy/effectiveness of 45% in the first 5 years and 0% in the following years.

^4^ Best scenario–High vaccine efficacy/effectiveness, high hospitalization rate, high lethality rate.

^5^ Worst scenario—Low vaccine efficacy/effectiveness, low hospitalization rate, low lethality rate.

When the model structure was changed and the vaccine effectiveness was 0% against pneumonia, the ICER had an increase of 873% (USD$1,297 to USD$12,625). When the "all-cause pneumonia" health state was replaced by the "pneumococcal pneumonia" state in the model with vaccine effectiveness of 45%, the ICER had an increase of 835% (USD$1,297 to USD$12,137), and in both analyses, the universal vaccination strategy would be cost-effective in both analytical perspectives.

When the price of vaccine dose was lower by 50%, the universal immunization strategy became cost-saving in the societal perspective. When the highest values of hospitalization costs (90^th^ percentile) were considered, the universal immunization strategy also became cost-saving from the societal perspective. The ICER also showed sensitivity to variations in hospitalization and mortality rates.

Overall, the univariate and structural sensitivity analysis demonstrated that changes in parameters resulted in ICER changes that would not change the conclusion that universal immunization of elderly with PPV23 is a cost-effective strategy in Brazil. Only in the worst scenario, very unlikely in the real world, the ICER (USD$48,995) was no longer cost-effective because it was above the cost-effectiveness threshold (USD$33,915).

## Discussion

This study evaluated the cost-effectiveness of introducing universal immunization of adults aged 60 years with PPV23 in Brazil, considering hospitalizations and deaths for IPD (meningitis, sepsis and other) and all-cause pneumonia and estimating burden of disease and costs based on secondary local data from local health information systems. The results of an economic evaluation of health interventions are commonly expressed as an ICER, the ratio of change in costs between two strategies to the change in effectiveness between the strategies: (cost strategy 1 –cost strategy 2) / (effectiveness strategy 1 –effectiveness strategy 2). The ICER provides the additional cost associated with the additional benefit of the new intervention being evaluated. Therefore, may offer to policy makers information on where resources should be allocated when they are scarce.

Our base case scenario, considered vaccine efficacy for IPD of 68%, in the first two years after vaccination, with waning of 6.8% and 8.5% in the 3^rd^ and 5^th^ year and 25% for all-cause pneumonia in the first five years, resulted in ICERs of USD$1,297 and USD$904 per LYS from health system and societal perspective, respectively. The intervention is very cost-effective, considering WHO criteria and 2011 Brazilian GDP per capita of USD$11,305.

Another study, which analyzed elderly universal vaccination with PPV23 in Brazil, including only pneumococcal pneumonias (bacteremic and non-bacteremic) as clinical outcomes, found the intervention cost-effective from the health system perspective and cost-saving from the societal perspective [18] but comparing and interpreting results of different cost-effectiveness studies must be done with care, given the different methodological strategies, assumptions and sponsors.

We choose life years as primary outcome because it is an easy and transparent method for measuring population health, and there are few value choices involved. Only mortality was quantified and included in denominator of our incremental cost-effectiveness ratio. As we did not consider morbidity related to meningitis sequelae, we probably biased the results against the new technology (PPV23). We preferred to take this conservative approach instead of using QALYs. In Brazil, utility weights are still not available. Then, utilities would need to be obtained from other countries, which would not be transferable to the Brazilian setting. We preferred to be conservative and did not include quality of life in our analysis. Doing that, we avoided introducing a parameter subject to a great deal of uncertainty into the model and did not bias the cost-effectiveness ratio in favor of the PPV23.

In our study, the sensitivity analyses showed the PPV23 effectiveness, for which there is great uncertainty in literature, as the parameter that most affected the ICERs. However, considering low vaccine efficacy/effectiveness (53% for IPD in the first two years following vaccination, with waning of 6.8% and 8.5% in the 3^rd^ and 5^th^ year, respectively, and 2% for all-cause pneumonia in the first five years), the universal immunization program would still be cost-effective from both analytical perspectives. Taking into account the controversy regarding vaccine effectiveness in preventing pneumonias, in the sensitivity analyses we also considered that PPV23 protects just against IPD (vaccine effectiveness = 0% in preventing pneumonia). This was a conservative analysis, since it did not incorporate bacteremic pneumococcal pneumonia into IPD probability, and still the universal immunization would be considered cost-effective.

The ICERs were sensitive to hospitalization and case-fatality rates, as well as to hospitalization costs. Increase in these parameters resulted in reduction in the ICERs. Underdiagnosing / underreporting may be an issue when estimating burden of PD based on health information systems database. The burden of disease was probably underestimated in this study, considering underreporting to SINAN (meningitis) and medical practices. In Brazil, blood cultures are usually limited to severe-ill hospitalized patients and previous use of antibiotics before sample collection may affect the sensitivity of diagnosis. Case-fatality rates may have been underestimated since only hospital deaths were taken into account. Additionally, costs of disease were also probably underestimated, since we assumed the SUS costs for the private health system due to lack of data on hospitalization costs in the private sector.

Pneumococcal meningitis is frequently associated with the highest rates of neurological sequelae among adults [23, 24]. Data on neurological sequelae following meningitis among adults are scarce in Brazil [25]. Costs of treatment of sequelae are not available in the country and so, these costs were not included in our model, which also underestimates the costs of disease, resulting in a conservative analysis.

We did not consider pneumonia treated in outpatient care that constitutes a large part of all-cause pneumonia. Data on outpatient care by diagnosis is not available in the Brazilian Health Information Systems. Furthermore, data on PPV23 effectiveness in preventing pneumonia refers mainly to hospitalized cases. There is no reliable data on vaccine effectiveness in preventing outpatient pneumonia and including it in the model would increase uncertainty.

Since 1999, the Brazilian NIP has recommended PPV23 immunization for institutionalized elderly and persons over-two years with underlying conditions, but data on vaccine coverage in these populations is scarce. In 2011, 165,825 PPV23 doses were administered to adults aged >60 years. At US$8.46 per administered dose, the current program costs USD$1,403,338. Since we were not able to build a reliable estimate of the number of doses administered to adults aged 60 years (the cohort included in this evaluation), the costs of the current strategy was not included in the model, which also resulted in a conservative analyses, unfavorable to the PPV23 universal immunization program.

PPV23 price also impacted the ICERs in the sensitivity analysis. A 50% reduction in the price of vaccine dose would make the program cost-saving.

In high-income countries, the introduction of pneumococcal conjugate vaccines into universal childhood immunization led to reduction in PD caused by vaccine serotypes in the elderly (indirect protection) [26]. Recent studies have demonstrated vaccine effectiveness and impact of the Brazilian PCV10 childhood vaccination program in the target population [27–29], but indirect protection in other age groups has not been demonstrated yet. PCV10 has been introduced into universal childhood immunization in 2010 and indirect protection may take longer to be observed. Although indirect protection would generally be expected in populations with high childhood PCV coverage, it is difficult to predict the exact epidemiological effect of the vaccine in new settings, due to variation in the circulating serotypes and programmatic differences, such as the schedule used (number of doses and age of vaccination), catchup and vaccine coverage [30]. Surveillance of the burden of PD in the entire population and circulating pneumococcus serotypes is critical to evaluate the indirect effects of childhood immunization in adults aged >60 years. Our model did not consider indirect effects of childhood immunization in the burden of PD in adults aged >60 years. This methodological approach may have overestimated the effects of PPV23 immunization program.

The Advisory Committee on Immunization Practices has recently recommended sequential PCV13-PPV23 vaccination for all adults aged >65 years in USA [31]. Estimating cost-effectiveness of such strategy is challenging. Cost-effectiveness models require explicit assumptions of effectiveness to be specified, but clinical data on the efficacy of the sequential PCV13-PPV23 vaccination strategy are not available. No trials with clinical endpoints comparing PCV13 and PPV23 vaccines in adults are available either. Furthermore, the efficacy/effectiveness of PCV13 against IPV or non-bacteremic pneumococcal pneumonia in adults has not been demonstrated yet. There is no known correlate of protection, i.e. the antibody level at which clinical protection would be expected, for pneumococcal disease in adults. It is not possible to translate immunogenicity measures into expected efficacy/effectiveness. Results of published cost-effectiveness studies that compared different strategies of pneumococcal vaccination in adults were sensitive to effectiveness assumptions for each vaccine [32–35].

We concluded that universal immunization of persons aged 60 years with PPV23 is a very cost-effective strategy when compared with current practice in Brazil. Even when the model did not incorporate bacteremic pneumococcal pneumonia into IPD estimative, the universal immunization still would be considered cost-effective.
